# Supplementary material for: Pre-existing bilayer stresses modulate triglyceride accumulation in the ER versus lipid droplets
Source: eLife. 2021 Feb 1;10:e62886. doi: 10.7554/eLife.62886 (PMC7895522; doi:10.7554/eLife.62886)
Supplement: Supplementary file 1. — Supplementary table 1. Non-bonded parameters derived for this study. Supplementary table 2. Calculated values of πCH for each bilayer composition and the relative percentage change with respect to the composition ‘100% DOPC’. Supplementary table 3. Calculated values of κb for each bilayer composition with two different methods: from the real-space analysis of the instantaneous surface deformations (κb ReSIS) and from the Fourier-space analysis of the membrane fluctuations (κb Fluct). Supplementary table 4: S. cerevisiae strains used in this study. [file elife-62886-supp1.docx]

Supplementary file 1 for

**Pre-existing bilayer stresses modulate triglyceride accumulation in the ER *versus* lipid droplets.**

Valeria Zoni^1†^, Rasha Khaddaj^1†^, Pablo Campomanes^1^, Abdou Rachid Thiam^2^, Roger Schneiter^1^ and Stefano Vanni^1,^*

^1^  University of Fribourg, Department of Biology, 1700 Fribourg, Switzerland

^2^ Laboratoire de Physique de l’École Normale Supérieure, ENS, Université  PSL, CNRS, Sorbonne Université , Université de Paris, Paris, France

† These authors contributed equally.

Supplementary table 1: Non-bonded parameters derived for this study

| Interaction | LJ type | ε (kcal mol-1) | σ(Å) |
| --- | --- | --- | --- |
| OG-NH | LJ 9-6 | 1.0082 | 3.6925 |
| OG-PHE | LJ 9-6 | 1.8671 | 3.6925 |
| OG-OAB | LJ 9-6 | 0.4719 | 3.2325 |
| OG-C2T | LJ 9-6 | 0.2780 | 3.6340 |
| OG-CM2 | LJ 9-6 | 0.3310 | 3.5470 |
| OG-CM2R | LJ 9-6 | 0.3310 | 3.5470 |
| OG-CMDB | LJ 9-6 | 0.6970 | 3.4800 |
| OG-CMB | LJ 9-6 | 0.2780 | 3.6340 |
| OG-CMR | LJ 9-6 | 0.2780 | 3.6340 |
| OG-CMR5 | LJ 9-6 | 0.2780 | 3.6340 |
| OG-CTB | LJ 9-6 | 0.2780 | 3.6340 |
| OG-CTBA | LJ 9-6 | 0.3310 | 3.5470 |
| OG-CTBB | LJ 9-6 | 0.3310 | 3.5470 |

Supplementary table 2: Calculated values of π_CH_ for each bilayer composition and the relative percentage change with respect to the composition “100% DOPC”

| Bilayer composition | π_CH_ (mN/m) | Relative change |
| --- | --- | --- |
| 100% DOPC | 26.29 | 0% |
| +20 mol% DAG | 29.80 | +13.4% |
| +30 mol% DOPE | 29.59 | +12.6% |
| +10 mol% CHOL | 29.52 | +12.3% |
| +20 mol% DOPE | 29.13 | +10.8% |
| +10 mol% DAG | 28.51 | +8.4% |
| +60 mol% DLPE | 27.44 | +4.4% |
| +60 mol% DPPC | 23.66 | -10.0% |
| +60 mol% DLPC | 19.98 | -24.0% |

Supplementary table 3: Calculated values of *κ_b_* for each bilayer composition with two different methods: from the real-space analysis of the instantaneous surface deformations (*κ_b_* ReSIS) and from the Fourier-space analysis of the membrane fluctuations (*κ_b_* Fluct)

| Bilayer composition | *κ_b_* ReSIS (K_B_T) | *κ_b_* Fluct (K_B_T) |
| --- | --- | --- |
| 100% DOPC | 10.81 | 12.86 |
| +20 mol% DAG | 10.22 | 10.57 |
| +30 mol% DOPE | 11.45 | 11.55 |
| +10 mol% CHOL | 11.08 | 12.65 |
| +20 mol% DOPE | 11.17 | 10.87 |
| +10 mol% DAG | 10.57 | 10.20 |
| +60 mol% DLPE | 12.94 | 10.09 |
| +60 mol% DPPC | 13.11 | 13.36 |
| +60 mol% DLPC | 11.56 | 10.81 |

Supplementary table 4: *S. cerevisiae* strains used in this study

| **Strain** | **Relevant Genotype** | **Source** |
| --- | --- | --- |
| BY4741 | MaTa his3∆1 leu2∆0 met15∆0 ura3∆0 | Lab collection |
| RSY 3091 | Mat𝛂 his3∆1 leu2∆0 lys2∆0 ura3∆0 met15∆0 ole1ts | Jacquier et al 2010 |
| RSY 3077 | Mat𝛂 his3∆1 leu2∆0 lys2∆0 ura3∆0 met15∆0 are1::KanMX | Jacquier et al 2013 |
|  | are2::kanMX trp1::URA lro1::TRP dga1::Lox-HIS-Lox |  |
| RSY 5165 | Mat𝛂 his3∆1 leu2∆0 lys2∆0 ura3∆0 met15∆0 | Lab Collection |
|  | pah1:: KanMX are1::HIS3 are2::LEU2 |  |
| RSY 1023 | Mat𝛂 elo1::HIS3 leu2-2 112 can1-100 ade2-1 ura3-1 his3-11 | Lab collection |
